# Optimization of florfenicol dose against *Piscirickettsia salmonis* in *Salmo salar* through PK/PD studies

**DOI:** 10.1371/journal.pone.0215174

**Published:** 2019-05-13

**Authors:** Betty San Martín, Marcela Fresno, Javiera Cornejo, Marcos Godoy, Rolando Ibarra, Roberto Vidal, Marcelo Araneda, Arturo Anadón, Lisette Lapierre

**Affiliations:** 1 Laboratorio de Farmacología Veterinaria, Departamento de Ciencias Clínicas, Facultad de Ciencias Veterinarias y Pecuarias, Universidad de Chile, Santiago, Chile; 2 Laboratorio de Inocuidad Alimentaria, Departamento de Medicina Preventiva, Facultad de Ciencias Veterinarias y Pecuarias, Universidad de Chile, Santiago, Chile; 3 Centro de Investigaciones Biológicas Aplicadas (CIBA), Puerto Montt, Chile; 4 Facultad de Medicina Veterinaria, Universidad San Sebastian, Puerto Montt, Chile; 5 Instituto Tecnológico del Salmón, Puerto Montt, Chile; 6 Facultad de Medicina, Universidad de Chile, Santiago, Chile; 7 Benchmark Genetics Chile, Puerto Montt, Chile; 8 Departamento de Farmacología y Toxicología, Facultad de Veterinaria, Universidad Complutense de Madrid, Madrid, Spain; Cornell University, UNITED STATES

## Abstract

Salmonid Rickettsial Septicemia (SRS) is the disease of greatest economic importance in the Chilean salmon farming industry, causing high mortality in fish during the final stage of their productive cycle at sea. Since current, commercially available vaccines have not demonstrated the expected efficacy levels, antimicrobials, most commonly florfenicol, are still the main resource for the treatment and control of this pathogen. The aim of this study was to determine the most appropriate single dose of florfenicol, administered through medicated feed, for the treatment of *Piscirickettsia salmonis (P*. *salmonis*), using pharmacokinetic/pharmacodynamic (PK/PD) models. Previously, Minimum Inhibitory Concentrations (MICs) of florfenicol were determined for 87 *P*. *salmonis* isolates in order to define the epidemiological cut-off point (CO_WT_). The most commonly observed MIC was 0.125 μg mL^-1^ (83.7%). The CO_WT_ value was 0.25 μg mL^-1^ with a standard deviation of 0.47 log_2_ μg mL^-1^ and 0.36 log_2_ μg mL^-1^, for Normalized resistance interpretation (NRI) method and ECOFFinder method, respectively. A MIC of 1 μg mL^-1^ was considered the pharmacodynamic value (PD) to define PK/PD indices. Three doses of florfenicol were evaluated in fish farmed under controlled conditions. For each dose, 150 fish were used and blood plasma samples were collected at different time points (0–48 hours). PK parameters were obtained from curves representing plasma concentrations as a function of time. The results of Monte Carlo simulation indicate that at a dose of 20 mg/Kg l.w. of florfenicol, administered orally as medicated feed, there is 100% probability (PTA) of achieving the desired efficacy (AUC_0-24h_/MIC>125). According to these results, we suggest that at the indicated dose, the PK/PD cut-off point for florfenicol versus *P*. *salmonis* could be 2 μg mL^-1^ (PTA = 99%). In order to assess the indicated dose in Atlantic salmon, fish were inoculated with *P*. *salmonis* LF-89 strain and then treated with the optimized dose of florfenicol, 20 mg/Kg bw for 15 days.

## Introduction

Resistance to antimicrobials is a common problem in human and veterinary medicine, therefore the World Organization for Animal Health [1], jointly with FAO/WHO, points out that preventative measures should be carried out under the "One Health" approach; thus promoting the responsible and prudent use of antimicrobials in the human population as well as in terrestrial and aquatic animals.

*Piscirickettsia salmonis* is a gram-negative bacterium, facultative intracellular, aerobic, pleomorphic, not encapsulated and is the etiological agent of piscirickettsiosis or Salmonid Rickettsial Septicemia (SRS) [2, 3]. It has affected the Chilean salmon farming industry since 1989 and is characterized by causing high mortality in fish at the fattening stage, the final stage of the productive cycle at sea. It is the most important disease of in the salmon-farming sector in terms of economic impact, generating economic losses of up to US $450 million, due to mortality, antibiotic treatment and vaccination costs [3, 4].

Since current, commercially available vaccines have not demonstrated the expected efficacy, antimicrobials are still the main resource for the treatment and control of this pathogen [3]. According to information provided by the Program for Sanitary Management of National Fisheries and Aquaculture Services of Chile [5], around 90% of antimicrobial therapies carried out in seawater farming sites are aimed at the treatment of Piscirickettsiosis, with florfenicol being the most common line of defense. This report also states that veterinarian-recommended doses of florfenicol for oral therapies range from 20 to 40 mg/kg l.w., even though the Veterinary Medical Registry of Chile recommends a dose of 10 mg/kg l.w. for various salmonid species. This is a major concern for both the salmon industry and national authorities, as studies indicating the most appropriate dose of florfenicol in salmonids are lacking.

Florfenicol is a broad-spectrum antimicrobial used in veterinary medicine that belongs to a family of agents that includes thiamphenicol and chloramphenicol [6]. Florfenicol is a molecule of synthetic origin; it is a structural analog of thiamphenicol and has greater *in vitro* activity against pathogenic bacteria than chloramphenicol and thiamphenicol [7, 8]. The phenicol group acts to inhibit bacterial protein synthesis by binding to the 50S and 70S subunits in the ribosome, abolishing peptidyl transferase activity [9]. Florfenicol is not susceptible to inactivation by chloramphenicol transacetylase. It was approved in the European Union in 1995 for use in veterinary medicine for the control and treatment of several bacterial diseases in cattle, pigs and later in commercial salmon farming [8]. It is now widely used in aquaculture for the treatment of different pathogenic bacteria in fish, including *Aeromonas salmonicida*, *Aeromonas hydrophila*, *Vibrio anguillarum*, *Vibrio salmonicida*, *Edwardsiella tarda*, *Edwardsiella ictaluri*, *Flavobacterium psychrophilum*, *Flavobacterium columnare* [10–13]. Florfenicol was licensed in Chile in 1994 to treat diverse bacterial diseases in Chilean salmon farming [13]. The recommended dosage of florfenicol is usually 10 mg/Kg bw once daily for 10 days [10, 14–17], but some authors recommend 15 mg/Kg bw for 10 consecutive days [18, 19]. Florfenicol is effective against intracellular microorganisms, like *Piscirickettsia salmonis* [20–21], as it is highly lipophilic, provides concentrations high enough to treat intracellular pathogens and to cross some anatomic barriers and is primarily bacteriostatic. Reports on pharmacokinetic profiles of florfenicol in different animal species indicate that its bioavailability is close to 90%, achieving high concentrations in plasma and peripheral tissues due to its low binding to plasma proteins [6, 22–24]. Pharmacokinetic profiles have also been described for a variety of fish species, including Atlantic salmon [25–29], which consistently demonstrate high bioavailability, high volume of distribution and rapid elimination.

In recent years, there have been major changes in the dosing regimens of existing antimicrobials, as an "optimal antibiotic therapy" must achieve bacterial eradication and resolution of the infection with minimal impact on the development of resistance [30]. This requires integrating knowledge of the mechanisms involved in the effect of antimicrobials (pharmacodynamics) and the evolution of antimicrobial concentrations in the organism (pharmacokinetics) through pharmacokinetic/pharmacodynamic models (PK/PD) [31]. Therefore, the integration of PK/PD into dosage-regimen optimization is of critical interest. In these PK/PD models, the pharmacodynamic parameter (PD) commonly used is the Minimum Inhibitory Concentration (MIC), defined as the lowest concentration of an antimicrobial that visibly inhibits bacterial growth after an incubation period under standardized conditions [32].

Several authors have used validated and standardized protocols to determine the MIC values of florfenicol against isolated *P*. *salmonis* obtained from different marine farms in different periods [20, 21, 33]. These studies describe MIC ranges for wild-type strains (WT) and epidemiological cut-off points (CO_WT_).

On the other hand, because florfenicol has been shown to be an independent concentration-antimicrobial with prolonged post-antimicrobial effects, the most suitable PK/PD index for determining the optimal dose is AUC_0-24h_/MIC [4], as demonstrated in other animal species against *Streptococcus suis* [34], *Actinobacillus pleuropneumoniae* and *Pasteurella multocida* [35].

Due to the scarcity of information on the optimal dose of florfenicol in aquatic species, the aim of this study was to determine the most appropriate dose of this antimicrobial for the treatment of *Piscirickettsia salmonis* in *Salmo salar*, using PK/PD models to ensure its efficacy and to minimize development of resistance. To obtain the PD parameter, florfenicol MICs were evaluated in *P*. *salmonis* isolates obtained from marine farms located in different geographical zones of southern Chile. After assessing whether MIC ranges had a statistically normal distribution, the wild-type epidemiological cut-off point (CO_WT_) was determined.

To obtain PK parameters, three doses of florfenicol were evaluated in fish farmed under controlled conditions, in order to obtain a curve of plasma concentrations as a function of time. From each curve, the following PK parameters were obtained: AUC_0-24h_, Cmax and Tmax. The PK/PD surrogate established for each dose was: AUC_0-24h_/ MIC.

Based on Monte Carlo simulation, the dose with a probability greater than 90% of reaching the pharmacodynamic objective (PTA) is defined using the PK/PD indices obtained in each work group. PTA values > 90% are considered indicative of efficacy according to Canut *et al*. (2015) [30] and Turnidge and Paterson (2007) [36].

In order to assess the optimized dose of florfenicol, in the control of *P*. *salmonis* in Atlantic salmon, a challenge assay was performed under controlled conditions.

## Material and methods

### Bacterial isolation and identification

A total of 87 *Piscirickettsia salmonis* isolates, obtained from fish infected with Salmonid Rickettsial Septicemia (SRS) at different aquaculture farms in southern Chile, between south latitude -41,8159 and 45,8159, and west longitude -73,1134 and -73,4931, with an average temperature between 8 and 16°C, and a density between 1 and 25 Kg/m^3^ were analyzed. Eighty-three strains were isolated between 2014 and 2017, and 4 were isolated between 2012 and 2013. Diagnosis was made by the veterinarian responsible for the aquaculture farms based on clinical signs, results of necropsy (lesions on the liver, skin, muscles, etc.) and RT-PCR for *P*. *salmonis*, according to the recommendations of Karatas e*t al*, 2008 [37].

For bacterial isolation, samples were taken with a sterile handle directly from those organs showing macroscopic signology consistent with SRS, such as nodular lesions on the liver, vesicles on the skin, bullae, ulcers, muscle caverns, splenomegaly, hepatomegaly and congestive enteritis, among others [38, 39]. Samples were inoculated using the stria method on *Piscirickettsia salmonis* agar plates (PSA) for subsequent incubation at 16°C for 6 to 12 days [40].

Bacterial identification was performed by Gram stain, immunofluorescence and polymerase chain reaction (PCR) according to the protocol described by Karatas *et al*. (2008) [37]. The isolates identified as *P*. *salmonis* were placed in cryovials (Cryobank Copan) following the manufacturer's recommendations; they contained a mixture of 80% AUSTRAL-SRS broth [41] and 20% DMSO (Merck), and were frozen at a temperature of -80°C.

### Determination of the Minimum Inhibitory Concentration (MIC)

The MIC values of florfenicol against *P*. *salmonis* were determined using the Broth Microdilution Technique following the recommendations of the Clinical and Laboratory Standards Institute [42, 43].

Isolates stored in the cryovials were seeded on CHAB Agar plates (heart infusion broth, 25 g L^-1^, glucose 10 g L^-1^, L-cysteine 1 g L^-1^, agar 15 g L^-1^, hemoglobin 2 g L^-1^, supplemented with 5% sheep blood), which were incubated at 16° C for 7 to 12 days [39]. At the end of the incubation period, bacterial inoculum was prepared at a concentration of 0.5 McFarland, using the AUSTRAL SRS broth described by Yañez *et al*. (2012) [41]. Concentration was corroborated by spectrophotometry using a Thermo Fisher Scientific certified standard.

Stock solutions of 1,280 μg/mL of florfenicol (Dr. Ehrenstorfer) were prepared using absolute methanol as a solvent. Stock solutions were protected from light and stored in aliquots of 1 mL at -80 ± 2°C, in order to maintain the stability of the antimicrobial. At the time MIC analysis, two-fold serial dilutions were performed, obtaining concentrations ranging from 0.0156 to 128 μg mL^-1^.

To each well 160 μL of AUSTRAL-SRS broth + 20 μL of antimicrobial and 20 μL of bacterial inoculum were added. A well without antimicrobial inoculated with the reference strain *P*. *salmonis* LF89 (ATCC VR-1361) was used as a positive control and a well with AUSTRAL-SRS broth without antimicrobial or bacterial inoculum was used as a negative control. All wells were incubated for 10 days at 16°C under gentle agitation.

The MIC value was determined by absorbance, using a Pharo 300 spectrophotometer (Spectroquant), calibrated at a wavelength of 625 nm. The absorbance of each well was compared to the negative control without bacterial inoculum (absorbance equivalent to 0). All experiments were performed in triplicate.

### Determination of epidemiological cut-off points (CO_WT_)

The epidemiological cut-off points (CO_WT_) were calculated using the following two available analytical methods:

- ECOFFinder, which is based on the methodology described by Turnidge *et al*. (2006) [36], where a normal logarithmic distribution is adjusted to the presumptive count of WT strains by means of iterative statistical methods. The configuration used was 99.9%.
- Normalized Resistance Interpretation (NRI), which was developed based on the methodology described by Kronvall (2010) [44], where a mathematical reconstruction of the ideal peak of the distribution of the bacterial population is carried out. The configuration used was 97.5%. The NRI method was used with permission from the patent holder, Bioscand AB, TÄBY, Sweden (European patent No 1383913, US Patent No. 7,465,559).

To evaluate the accuracy of the results obtained by both methods, the standard deviation of 1.2 log_2_ μg mL^-1^, described by the NRI, was considered.

### Determination of pharmacokinetic parameters

#### Fish population

Four hundred and fifty Atlantic salmon (*Salmo salar*) with an average weight of 1000 grams were used. Fish were obtained from stock maintained by the experimental center since March 2017. The origin of the animals was STH Center fish farm, Lot SNAQGS-216, Caliboro, Los Angeles, Chile. Fish were distributed and maintained in tanks of 1 m^3^ salt water per group (30 ppt) at 13 ± 0.5°C, with a flow of 120 L/min, which mimics commercial growing conditions. Ten tanks (15 fish/tank, average density per tank of 16 Kg/m^3^) were assigned to each of the following treatments: 1) Group 1: fed 10 mg florfenicol/Kg bw; 2) Group 2: fed 15 mg florfenicol/Kg bw; Group 3: fed 20 mg florfenicol/Kg bw. Daily, parameters of salinity, pH, temperature and oxygen, were measured to assure that these conditions ensure fish welfare. Prior to testing, the fish were given a 10-day acclimation period. During this period, they were given a feed ration corresponding to 0.8% of their body weight, using an antimicrobial-free commercial diet. The experiment lasted a total of 12 days, considering the acclimation period, administration of the treatment and sampling. There was no mortality during the trial. The fish were monitored and managed by a trained veterinarian who verified fish health and welfare daily.

#### Florfenicol medicated feed

Medicated feed of 6 mm caliber was produced by a commercial animal feed manufacturer (SalmoFood), using a premix of florfenicol 50% (Veterin 50%), according to the nutritional requirements of the fish under study, in a final concentration of 5 Kg of florfenicol per Ton of feed. Prior to the study, the concentration and homogeneity of florfenicol in the feed was corroborated using LC MS/MS chromatography. The Specific Feed Rate (SFR) for each group of fish was calculated, so that the medicated feed consumed would adjust to the dose required for each study. For each group, the following SFR was calculated: 1) Group 1: Dose: 10 mg/Kg bw, Average weight: 1400 g, 0.4% SFR; 2) Group 2: Dose: 15 mg/Kg bw, Average weight: 1000 g, 0.6% SFR; 3) Group 3: Dose: 20 mg/Kg bw, Average weight: 700 g, 0.8% SFR.

#### Treatment and sampling

Three doses of florfenicol were used (Group 1: 10 mg/Kg bw, Group 2: 15 mg/Kg bw, Group 3:20 mg/Kg bw) (Table 1). The medicated feed was administered in small quantities (micro-rations) over the course of the hours between 9:00 and 16:00 for one day, to ensure the full intake of the drug. At each time point, 12 fish from each tank were randomly selected for testing. After administration of medicated feed, blood plasma samples were taken between 0 and 48 hours, by caudal venipuncture at the following times: 0, 3, 6, 8, 12, 18, 24, 36 and 48 hours after the introduction of medicated feed. The samples were collected in EDTA tubes and centrifuged at 4500 rpm for 10 minutes. Plasma was collected after centrifugtation and stored at -20°C in microfuge tubes until analysis. After the sampling, all salmon were immediately euthanized by contusion, according to animal welfare recommendations from the VICH GL9 GCP [45], European Agency for the Evaluation of Medicinal Products [46], and Directive 2010/63/EU [47]. Necropsy was performed on all the fish, in order to verify the presence of feed in the stomach and their general health.

**Table 1:** Sampling times and tank distribution for each group.

Samples	T0	T1	T2	T3	T4	T5	T6	T7	T8	T9
**Tank number**	1	2	3	4	5	6	7	8	9	10
**Hours after feeding**	0	1	3	6	8	12	18	24	36	48
**Number of animals**	15	15	15	15	15	15	15	15	15	15

#### Chemical reagents and standards

A florfenicol standard provided by Dr. Ehrenstorfer GmbH (Formula C12H14NO4CI2SF, CAS N°73231-34-2, Augsburg, Germany) and a florfenicol amine standard obtained from Toronto Research Chemicals (Formula C10H15CIFNO3S, CAS N°108656-33-3, Toronto, Canada) were used. HPLC-grade methanol, HPLC-grade water, HPLC-grade acetonitrile and PA grade oxalic acid were purchased from Merck (Darmstadt, Germany).

#### Sample preparation

Extraction was carried out according to the method described by Li *et al*. (2006) [48], with modifications. Two hundred and fifty μl of plasma were placed in 50 mL tubes and 50 mL of 0.01 M oxalic acid dihydrate extraction solution in water/methanol 1:1 was added. The tubes were shaken for 10 minutes, sonicated for 20 minutes and centrifuged at 5,000 rpm for 5 minutes. Subsequently, 300 μl of the lower phase were transferred to a vial to be injected into the chromatographer. The limit of detection (LOD) and the limit of quantification (LOQ) established for both analytes in plasma were 0.1 μg/ml and 0.2 μg/ml, respectively.

#### Instrumental analysis

For instrumental analysis, an LC (Agilent, 1290 infinity series) coupled to a triple quadrupole mass spectrometer (API 5500, ABSCIEX) was used. A Synergi^TM^ 4 μm fusion RP 80Â 50 x 2.0 mm analytic column was used.

Analyst 1.6.3 and Multiquant 3.0 software was used for equipment management and integration, respectively. Chromatographic separation was performed through a mobile phase using solvent A: 0.1% of acetic acid in water; and a mobile phase solvent B: 0.1% of acetic acid in water/methanol 1:9 ratio; with a gradient flow of 350 μL min^-1^ and a gradient elution from 32% up to 68% solvent A in 3 min of 35% phase solvent A, and 75% phase solvent B. Injection volume was 2 μL; column oven temperature was set at 37°C. The mass spectrometer was operated according to the parameters listed in Table 2. The masses of the monitored ions are listed in Table 3.

**Table 2:** Parameters of the MS/MS detector.

Ionization	Electrospray (ESI)
**Scan type**	MRM
**Source temperature (TEM)**	550°C
**Nebulizer (GS1)**	60 psi
**Turbo ion (GS2)**	80 psi
**Curtain gas (CUR)**	20 psi
**Collision gas (CAD)**	10 psi
**Ion-spray voltage (IS)**	4500 V

**Table 3:** Monitored ion masses.

Analyte	Precursor ion (Q1 mass) (Da)	Fragment ion (Q3 mass) (Da)	Time (ms)	EP (V)	CE (V)	CXP (V)
**FF1**	356.0	336.0	100.0	-5,000	-15,000	-8,000
**FF2**	356.0	185.0	100.0	-5,000	-17,000	-12,000
**FFA1**	248.0	230.0	200.0	5,000	22,000	25,000
**FFA2**	248.0	130.0	200.0	2,000	30,000	10,000
**CAF-d5 (IS)**	326.0	157.0	100.0	10,000	-25,000	-20,000

#### Validation of analytical method

Prior to determining concentrations in plasma, the analytical method was validated by HPLC MS/MS, according to instructions from the European Commission Decision 2002/657/EC (2002). Precursor ions and the two product ions were identified for florfenicol and florfenicol amine, respectively. Values for essential parameters were estimated for the validation of the analytical method on plasma: specificity, recovery, repeatability, intralaboratory reproducibility, decision limit (CCα), detection capability (CCβ), and linearity.

#### Determination of PK/PD indices

In order to obtain the pharmacokinetic (PK) parameters, the plasma concentration curves are shown as a function of time. A one-way analysis of variance (ANOVA) and a Kruskall-Wallis test were performed. The AUC and Cmax and Tmax pharmacokinetic parameters were calculated with the Phoenix WinNonlin software (Version 8.0, Pharsight Corporation, Mountain View, CA, USA) using non-compartmental analysis. The PK/PD index for each dose was AUC_0-24h_/MIC. The MIC was 2 dilutions higher than the calculated CO_WT_.

#### Determination of the florfenicol dose

We performed a Montecarlo simulation based on the PK/PD indices defined for each dose and 6 minimum inhibitory concentrations (0.06, 0.125, 0.25, 1, 2 and 4 μg mL). Each iteration enabled inclusion of a new value among the sources of uncertainty, which is generated through its own probabilistic function. The results were evaluated under the following objective: Reference Point (PRO); *PRO*_*IE*_ *≥* *125*. The Risk Simulator 2012 software (Real Options Valuation, Inc. 2005–2012) was used to adjust probability distributions; the number of iterations was 10,000.

Previously, for each group defined as a source of uncertainty, a probability function was estimated by evaluating a series of probabilistic density functions (parametric test). The Kolmogorov-Smirnov test was used as an evaluation and selection test. Table 4 shows the results of the adjustment, type of distribution and its parameters.

**Table 4:** Adjustment, type of distribution and parameters of the Kolmogorov-Smirnov test.

S. Uncertainty	Distribution	Parameter	Parameter	K-S (p)
**AUC10 mg**	Gumbel	159.8 (alfa)	38.1 (Beta)	p = 0.992
**AUC15 mg**	Gumbel	183.5 (alfa)	26.6 (Beta)	p = 0.950
**AUC 20 mg**	Gumbel	537.0 (Alfa)	38.8 (Beta)	p = 0.992

The most suitable dose was that which was most likely (PTA) to reach plasma concentrations over the MIC that were greater than 90%, according to Canut *et al*., 2015 [30].

For more information about the protocol “Determination of florfenicol and florfenicol amine in fish plasma (*Salmo salar*) through HPLC MS/MS” enter the following link: dx.doi.org/10.17504/protocols.io.zhdf326

### Evaluation of optimized dose of florfenicol for the control of *P*. *salmonis* in Atlantic salmon (*Salmo salar*)

#### Fish population

Two hundred and seventy Atlantic salmon (*Salmo salar*) with an average weight of 400 grams were used. Fish were obtained from stock maintained by the experimental center since April 2018. The origin of the animals was Aquasan SA, Rio Maullin fish farm, Lot S17AGBLURM, region X, Chile. Fish were distributed and maintained in tanks of 0.5 m^3^ salt water per group (30–33 ppt) at 14 ± 1°C, with a flow of 120 L/min, which mimics commercial growing conditions. Nine tanks (30 fish/tank, average density per tank of 23.7 Kg/m^3^) were assigned to each of the following treatments: 1) Determination of LD50: Dilution 1/10; 2) Determination of LD50: Dilution 1/100; 3) Determination of LD50: Dilution 1/1000; 4) Determination of LD50: Dilution 1/10000; 5) Challenge with *P*. *salmonis* (2 tanks); 6) Challenge with *P*. *salmonis*: No treatment (positive control, 2 tanks); 8) Not challenge with *P*. *salmonis*: No treatment (negative control). Daily, parameters of salinity, pH, temperature and oxygen, were measured to assure that these conditions ensure fish welfare. Prior to testing, the fish were given a 7-day acclimation period. During this period, they were given a feed ration corresponding to 0.8% of their body weight, using an antimicrobial-free commercial diet. The animals were examined by the Veterinarian of the experimental center, to confirm that the specimens were suitable for the study. The sanitary condition of these was approved through laboratory analysis in samples, through RT-PCR for IPN virus, *Piscirickettsia salmonis* and *Renibacterium salmoninarum*. The group was vaccinated in the center of origin with ALPHA JECT 5.1 and ALPHA JECT LiVacSRS vaccine, which mimics commercial growing conditions at sea.

The determination of LD50 lasted a total of 38 days, considering the acclimation period (7 days), challenge with *P*. *salmonis* (1 day) and monitoring of the fish (30 days). The challenge with *P*. *salmonis* lasted a total of 38 days, considering the acclimation (7 days), main challenge (1 day), administration of treatment (15 days) and monitoring of the fish after treatment (15 days). The fish were monitored and managed by a trained veterinarian, who verified fish health and welfare daily.

#### Determination of Lethal Dose 50 (LD50)

The *P*. *salmonis* standardized isolate (LF-89) was provided by ADL Diagnostic Chile Ltda. The mother inoculum registered a titre of 10^7.5^ TCID_50_/mL, determined through the Karber-Spearman method, from which dilutions were made by a factor of 10 (1/10, 1/100, 1/1000 and 1/10000) (Table 5). The purity of the isolate was evaluated, considering analysis of RT-PCR ISAv, IPNv, BKD, *F*. *psycrophilum* and bacterial cultures.

**Table 5:** LD_50_ bacterial counts of inoculum dilutions.

Inoculum dilution	Bacterial count (ufc/mL)
**1:10**	8.47 x 10°
**1:100**	7.10 x 10^7^
**1:1000**	7.33 x 10^6^
**1:10000**	4.80 x 10^5^

To conduct the challenge, fish were extracted from the tank and placed in a container with anesthetic solution (25 mL AQUI-S/100 L water, 1–2 minutes for deep sedation), then taken individually and held with the ventral side facing up. The needle was inserted at an angle of approximately 90° in the ventral midline, between the pectoral and pelvic fins, injecting 0.2 mL of the inoculum / fish. After inoculation, the specimens were transferred to their original tanks; their state of recovery was monitored constantly. In general, the fish were observed to be in good condition with normal behavior. There was no post-inoculation mortality. After the procedure, the tanks were monitored for 30 days, during which data on mortality, feeding and environmental parameters were collected. Mortality was removed daily; the weight at the time of death was recorded along with the dilution corresponding to each tank. Throughout this phase, environmental parameters of salinity (ppt), pH, temperature and oxygen were measured and recorded, according to experimental center routine. The average temperature was 14.5 ± 0.58, salinity 32 ppt, pH 7.0 to 7.1, and oxygen levels 100–120% saturation. The mortality was analyzed in the Antares S.A Laboratory, where anatomopathological observation and RT-PCR SRS confirmed cause of death.

#### Medicated feed

Medicated feed of 4 mm caliber was produced by a commercial animal feed manufacturer (SalmoFood), using a premix of florfenicol 50% (Veterin 50%), according to the nutritional requirements of the fish under study, in a final concentration of 3.2 Kg of florfenicol per Ton of feed, for a dose of 20 mg/Kg bw. Prior to the study, the concentration and homogeneity of florfenicol in the feed was corroborated using LC MS/MS chromatography.

#### Challenge with *P*. *salmonis*

Five tanks were used (30 fish/tank), with an acclimation period of 7 days prior to the challenge. During this period, fish were visually inspected, observing good behavior and absence of mortality. The temperature registered an average of 14.6 ± 0.31°C and feed consumption varied from 0.56 to 1.04% SFR between the tanks. After acclimation, the challenge was carried out by intraperitoneal injection. The specimens were extracted from their original tank and placed in a container with anesthetic solution (25 mL AQUI-S/100 L water, 1–2 minutes for deep sedation), then taken individually and inoculated with the same procedure described for the LD50 determination, using the *P*. *salmonis* standardized isolate (LF-89) with a dilution of 1/10. Fish samples were taken at days 3 and 6 post challenge, to be analyzed by anatomopathological observations and RT-PCR SRS (Laboratorio Antares S.A) to verify pathogen positivity. In this way, the presence of *P*. *salmonis* (kidney-liver and spleen matrix) was confirmed on day 3 post challenge, therefore treatment began on day 4.

#### Treatment with optimized dose of florfenicol

The medicated feed was administered in small quantities (micro-rations) over the course of the hours between 9:00 and 16:00 hours for 15 days, with a feed consumption of 1.25% SFR. Tanks with positive and negative controls were fed with an antimicrobial-free commercial diet. Feed consumption was estimated in all tanks and mortality was withdrawn daily and each time it occurred, the weight at the time of death was recorded along with the group corresponding to each tank. Mortality was analyzed in the Antares S.A Laboratory by anatomopathological observation and RT-PCR SRS to confirm cause of death. Throughout the challenge phase (treatment and subsequent monitoring), environmental parameters of salinity (ppt), pH, temperature and oxygen were measured and recorded, according to experimental center routine. The average temperature was 14.9 ± 0.32, salinity 32 ppt, pH 7.0 and oxygen levels 100–120% saturation. After the experiment, all salmon were inmediately euthanized by contusion, according to animal welfare recommendations from the VICH GL9 GCP [45], European Agency for the Evaluation of Medicinal Products [46], and Directive 2010/63/EU [47]. Necropsy was performed on all the fish in order to verify their general health and samples were taken from 2 fish per tank (kidney and liver) to confirm elimination of *P*. *salmonis* through RT-PCR SRS.

### Ethics statements

This study was carried out in accordance with the recommendations of the European Agency for VICH GL9 GCP [43], European Agency for the Evaluation of Medicinal Products [44], and Directive 2010/63/EU [45]. The protocol was approved by the “Institutional Committee for the care and use of animals (CICUA), Universidad de Chile”, approval numbers 17219-VET-UCH and 17221-VET-UCH on December 2017.

## Results

### Bacterial isolation and identification

Eighty-seven strains of *P*. *salmonis* were isolated and identified between 2012 and 2017. Seventy percent of the isolates were obtained in the last two years. Table 6 shows the distribution of these isolates by year, species and geographical area.

**Table 6:** Distribution of isolates by year, host species and geographical area.

		Number of isolates by	
Year	Total isolates	Species (n)	Geographic area (n)
**2012**	1	Atlantic salmon (*Salmo salar*) (1)	Chiloé (1)
**2013**	3	Atlantic salmon (*Salmo salar*) (2)	Chiloé (1)
			Melinka (1)
		Rainbow trout *(Oncorhynchus spp)* (1)	Chiloé (1)
**2014**	11	Atlantic salmon (*Salmo salar*) (10)	Melinka (3)
			Chiloé (6)
			Quellón (1)
		Rainbow trout *(Oncorhynchus spp)* (1)	Reloncaví Estuary (1)
**2015**	11	Atlantic salmon (*Salmo salar*) (11)	Chiloé (6)
			Calbuco (1)
			Puerto Aysén (1)
			Puerto Montt (3)
**2016**	33	Atlantic salmon (*Salmo salar*) (22)	Chiloé (4)
			Calbuco (2)
			Castro (1)
			Chaitén (2)
			Honopirén (1)
			Melinka (2)
			Puerto Aguirre (1)
			Puerto Aysén (6)
			Puerto Gala (1)
			Quellón (2)
		Coho salmon (*Oncorhynchus kisutch)* (9)	Calbuco (1)
			Chiloé (4)
			Honopirén (3)
			Quellón (1)
		Rainbow trout *(Oncorhynchus spp)* (2)	Chiloé (1)
			Puerto Aguirre (1)
**2017**	28	Atlantic salmon (*Salmo salar*) (22)	Puerto Gala (1)
			Chiloé (14)
			Calbuco (6)
			Quellón (1)
		Coho salmon *(Oncorhynchus spp)* (2)	Melinka (1)
			Hornopirén (1)
		Rainbow trout *(Oncorhynchus spp)* (4)	Puerto Aguirre (1)
			Puerto Aysén (1)
			Puerto Montt (1)
			Hornopirén (1)

### Distribution of MIC and determination of epidemiological cut-off points (CO_WT_)

MIC ranges of *P*. *salmonis* isolates *versus* florfenicol ranged from 0.06 to 0.25 μg mL^-1^. The most commonly observed MIC, representing 83.7% of all isolates, was 0.125 μg mL^-1^ (S1 Table).

**Fig 1:**
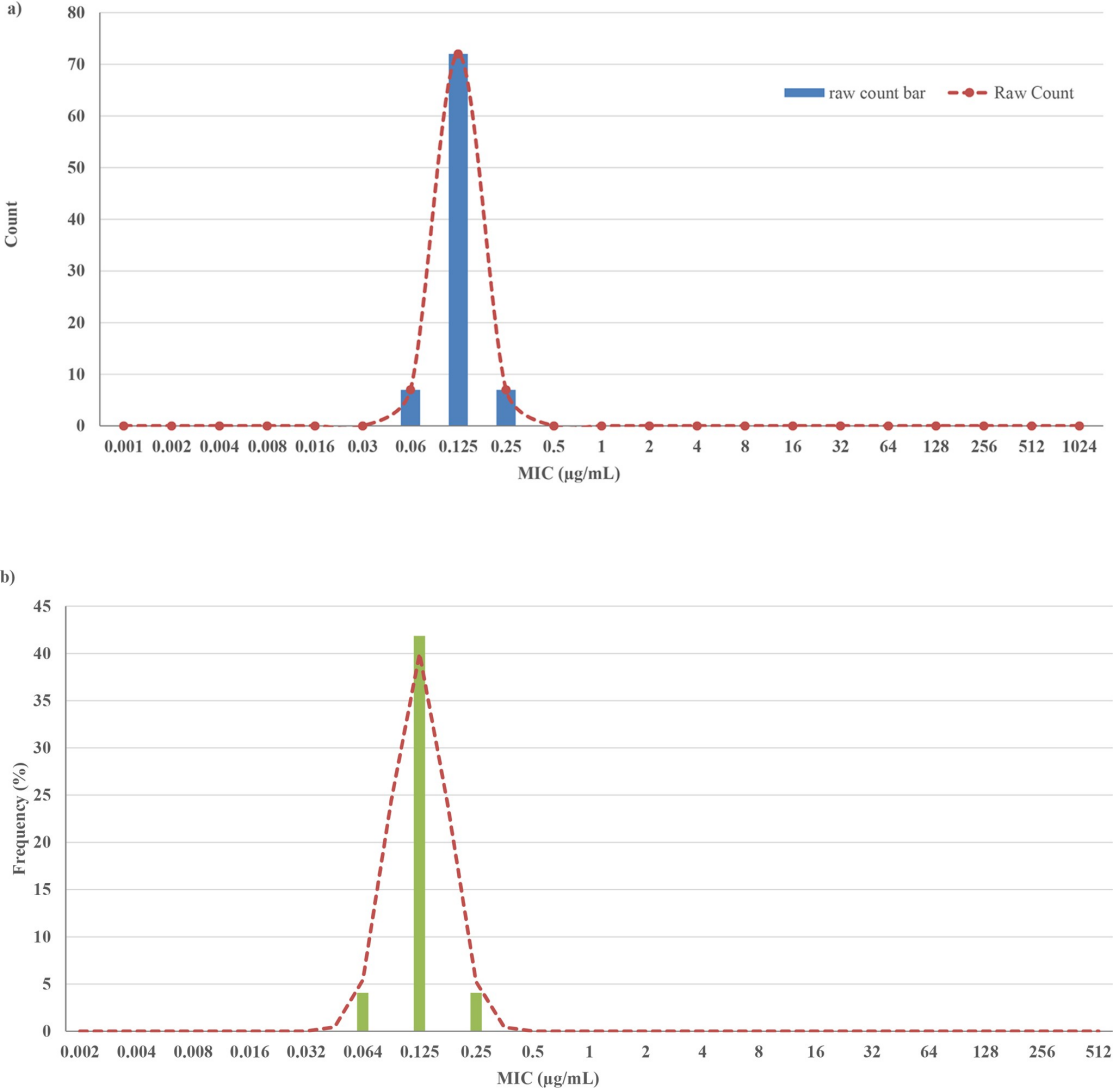
Distribution of the Minimum Inhibitory Concentration values of 87 strains of *Piscirickettsia salmonis* versu*s* florfenicol. Distributions obtained through the ECOFFinder (a) and NRI (b) programs. Bars represent the gross count of the number of isolates in each concentration of antimicrobial; lines represent the curves of best fit for the distribution of WT strains.

The CO_WT_ point was 0.25 μg mL^-1^ with a standard deviation of 0.47 log_2_ μg mL^-1^ and was calculated using NRI analysis. Data analysis using the ECOFFinder method provided the same point of CO_WT_ but with a lower standard deviation (0.36 log2 μg mL^-1^). Table 7 shows MICs and the CO_WT_ for florfenicol against isolates of *P*. *salmonis*. According to the results obtained, 100% of the isolates were classified as wild type (WT). The distribution of the MICs is shown in Fig 1.

**Table 7:** Minimum inhibitory concentration (MIC) and epidemiological cut-off points (CO_WT_) for the total strains of *P*. *salmonis* analyzed against Florfenicol.

		MIC (μg mL^-1^)	CO_WT_ (μg mL^-1^)			
Antibiotic	n	Range	NRI^1^	%WT	ECOFFinder^2^	%WT
**FF**	87	0.06–0.25	0.25	100	0.25	100

FF: Florfenicol

(^1^): Epidemiological cut-off point calculated by NRI

(^2^) Epidemiological cutoff point calculated using ECOFFinder; %WT: percentage of strains classified as WT.

### Determination of the PK/PD indices of florfenicol administered at different doses against *P*. *salmonis*

**Fig 2:**
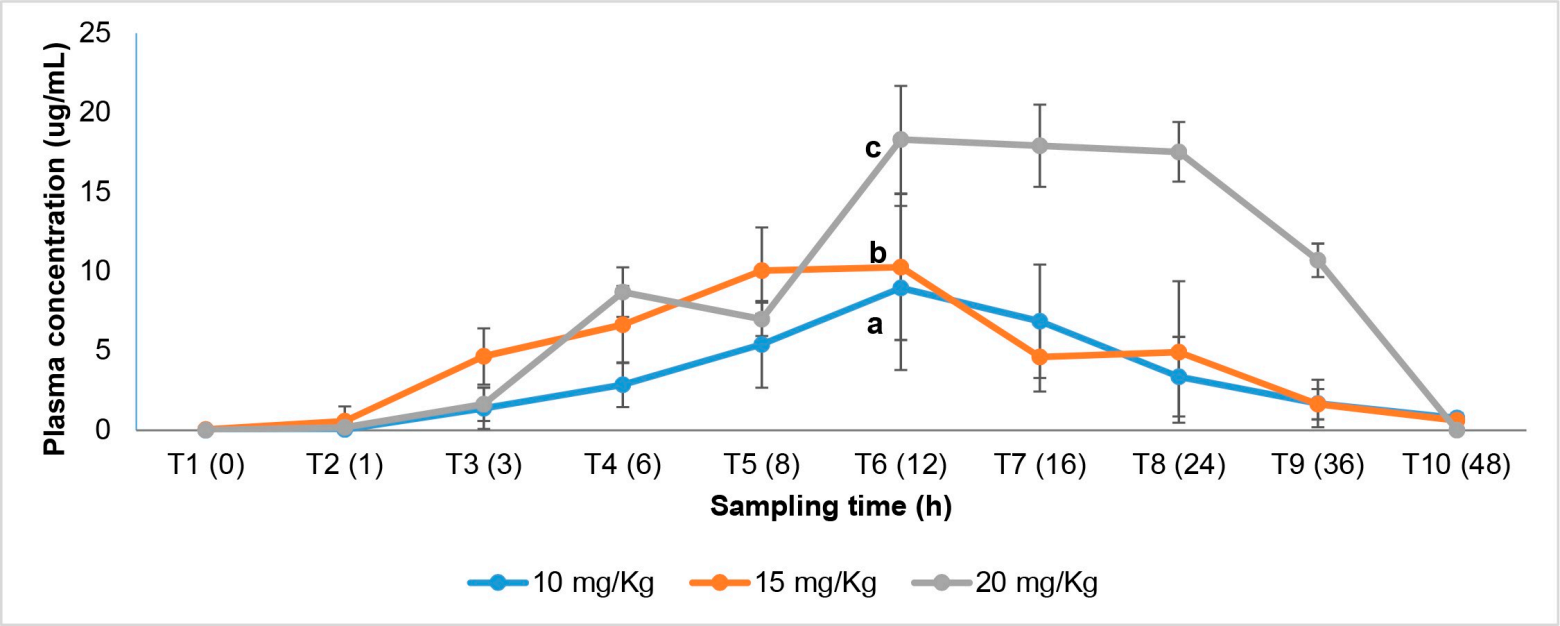
Average plasma concentrations (μg/mL) by sampling time (hours) and dose: 10, 15 and 20 mg/Kg. Different letters represent statistical differences between groups (p<0.05).

For each experimental group, the plasma concentrations of florfenicol were calculated based on the sum of florfenicol and florfenicol amine (active metabolite) (S2 Table). Fig 2 shows the plasma concentration curves as a function of time for each dose analyzed. The plasma concentrations reached for each dose were statistically different (p<0.05)., according to the one-way ANOVA and the Kruskall-Wallis test.

The pharmacokinetic parameters (PK) Cmax, AUC_0-24h_ and Tmax obtained from the curve of plasma concentrations, are shown in Table 8. For more information, see S3 Table.

**Table 8:** Average pharmacokinetics of florfenicol parameters by the trial dose.

Dose (mg/Kg)	AUC	Cmax (μg/mL)	Tmax (h)
**10**	141 ±45	11±3	13.3 ± 4.3
**15**	166 ±34	13 ±2.8	11±4.6
**20**	520±50	20±3	15±5

Considering that sensitivity studies are carried out in small populations, a MIC = 1 **μ**g mL^-1^ was considered the PD when defining PK/PD indices, which is equivalent to two dilutions higher than the CO_WT_ calculated in this work.

Table 9 shows the results of the AUC_0-24h_/MIC index for each dose studied.

**Table 9:** PK/PD index (AUC_0-24h_/ MIC) for doses 10, 15 and 20 mg/ Kg.

Dose (mg/Kg)	AUC_0-24h_/MIC (Average±DE)
**10**	141±45
**15**	166±34
**20**	520±50

### Definition of the most appropriate dose of florfenicol using the Montecarlo simulation

The statistical parameters and the probability of reaching a target reference point greater than 125 are shown in Table 10. The results of the Montecarlo simulation indicate that at a dose of 20 mg/Kg l.w. of florfenicol, administered orally through feed, there is a 100% probability (PTA) of achieving the desired efficacy (Table 11). At this dose, the 24-hour plasma concentrations (AUC_0-24h_) remained above the defined pharmacodynamic value (1 **μ**g mL^-1^). For more information, see S1 Fig.

**Table 10:** Statistical parameters and probability of reaching a target reference point greater than 125 (TRP>125) AUC_20 mg_/MIC_n_.

MIC	MIC = 0.06	MIC = 0.125	MIC = 0.250	MIC = 1	MIC = 2	MIC = 4
**Average**	8,580	4,118	2,059	515	257	129
**Median**	8,709	4,180	2,090	523	261	131
**Standard deviation**	824	395	198	49	25	12
**Percentile (25%)**	8,144	3.909	1.955	489	244	122
**Percentile (75%)**	9,162	4.397	2.199	550	275	137
**Probability (PRO>125)**	100.0%	100.0%	100.0%	100.0%	99.9%	68.3%

MIC: Minimum Inhibitory Concentration

**Table 11:** Integration of pharmacokinetic and pharmacodynamic variables (PK/PD) using the Montecarlo simulation.

Dose (mg/Kg)	MIC (μg/mL)	AUC_0-24h_/MIC (h ±SD)	PTA
**10**	0.06	2331±738	99.70
	0.125	1119±354	99.09
	0.25	559±177	97.92
	1	139±44	67.57
	2	70±22	0.00
	4	35±11	0.00
**15**	0.06	2805±563	99.94
	0.125	1346±270	99.87
	0.25	673±135	99.68
	1	168±33	89.58
	2	84±17	0.00
	4	42±8	0.00
**20**	0.06	8580±823	100.00
	0.125	4118±395	100.00
	0.25	616±115	100.00
	1	514±49	100.00
	2	257±24	99.99
	4	128±12	68.30

MIC: Minimum Inhibitory Concentration; SD: Standard deviation; PTA: Probability of reaching the objective AUC_0-24h_/MIC >125.

### Evaluation of optimized dose of florfenicol for the control of *P*. *salmonis* in Atlantic salmon (*Salmo salar*)

#### Determination of LD_50_

**Fig 3:**
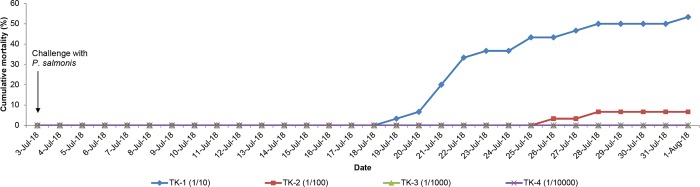
Cumulative mortality (%) in Atlantic salmon after *P*. *salmonis* challenge in dilutions of 1/10, 1/100, 1/1000 and 1/10000. TK: Tank.

Mortality was observed beginning on day 17 post challenge in the dilution with the highest concentration of the bacteria (1/10), and beginning on day 24 for the dilution of 1/100 (Fig 3). Dilutions of 1/1000 and 1/10000 did not show any mortality. According to these results, the dilution selected for the challenge was 1/10.

#### Challenge with *P*. *salmonis*

Samples analyzed by RT-PCR SRS, on day 3 post challenge presented a 75% prevalence of *P*. *salmonis* (Table 12). At necropsy, congestion was observed in adipose tissue and pyloric caeca, however, these findings are not entirely attributable to SRS. Samples analyzed at day 6 post challenge presented a 100% prevalence of *P*. *salmonis*. The findings in the necropsy were similar to those recorded on day 3, although greater congestion was observed in adipose tissue and pyloric caeca.

**Table 12:** RT-PCR SRS results at days 3 and 6 post challenge with *P*. *salmonis*.

Tank	Days post challenge	Fish (N°)	Ct	Prevalence SRS (%)
**2**	3	1	25.19	100
**2**	3	2	33.30	100
**3**	3	1	No Ct.	50
**3**	3	2	27.89	50
**5**	6	1	29.81	100
**5**	6	2	28.75	100
**6**	6	1	24.97	100
**6**	6	2	32.41	100

**Fig 4:**
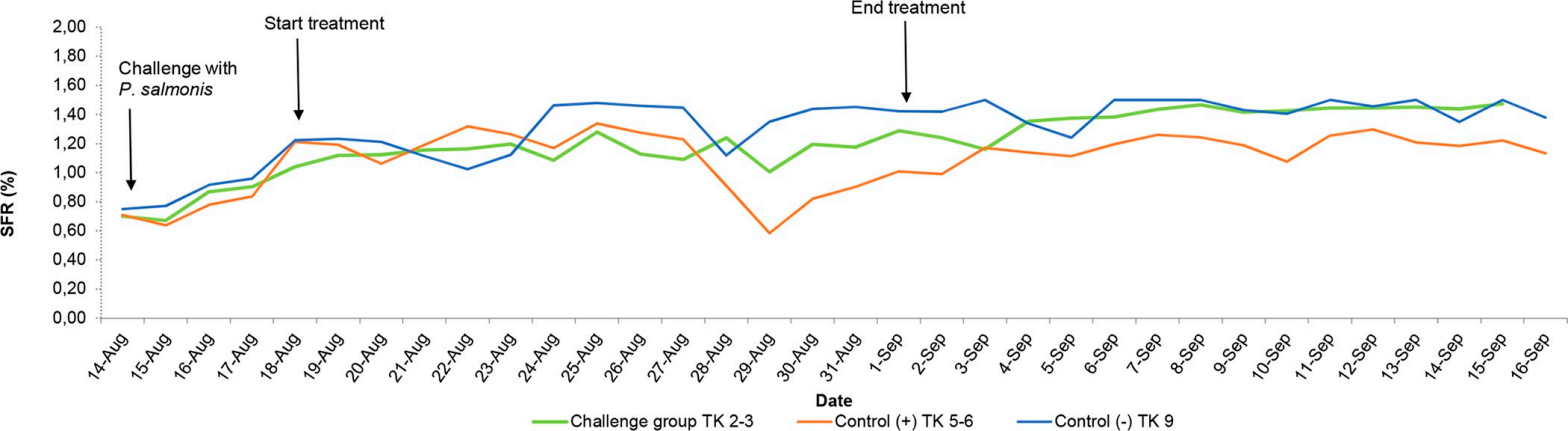
Specific Feed Rate (%) by date of experiment. Challenge group: Tanks 2 and 3; Control (+) (without medication): Tanks 5 and 6; Control (-) (without challenge): Tank 9. TK: Tank; SFR: Specific Feed Rate. For more information, see S4 Table.

**Fig 5:**
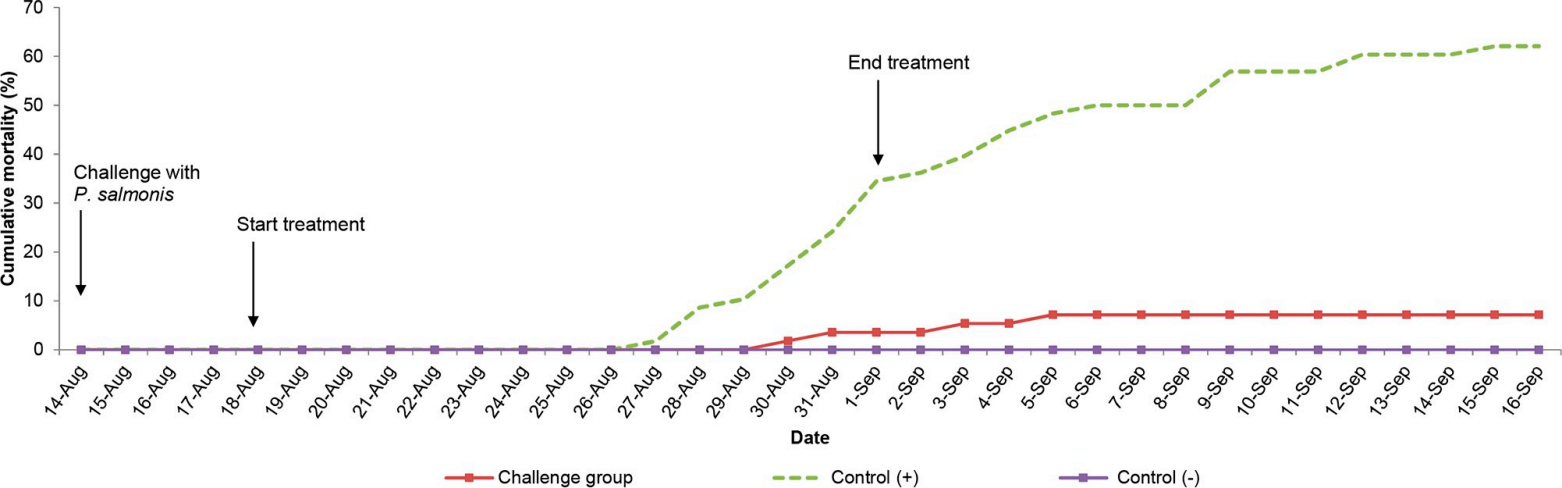
Cumulative mortality (%) by date of experiment. Challenge group: Tanks 2 and 3; Control (+) (without medication): Tanks 5 and 6; Control (-) (without challenge): Tank 9. For more information, see S5–S7 Tables.

In the challenged group, the fish maintained relatively constant feed consumption, with an average of 1.15% SFR in the 15 days of treatment. After treatment, the fish had an increase in feed intake, reaching SFR of 1.42 to 1.48% (Fig 4). This group presented low cumulative mortality, 7.14%, (Fig 5), with respect to the control group during the same period of time.

The positive control group, without antibiotic treatment, showed a decrease in feed consumption from the onset of mortality and throughout the outbreak. Subsequently, an increase in SFR consumption of 1.0 to 1.5%, which coincided with the stabilization of mortality, was observed. As can be seen, the control group without medication registered greater cumulative mortality compared to the group treated with florfenicol, reaching 63 and 64% in the respective tanks (Fig 5). With the exception of the first days of study, the negative control group maintained a constant feed consumption, with an average SFR of 1.36%. No mortality was recorded in this group (Fig 5).

All the anayzed mortality presented internal injuries attibutable to *P*. *salmonis*, with hepatomegaly, splenomegaly, hemorrhage in adipose tissue and pyloric caeca, and congestive brain being the most frequent. On the other hand, in all the analyzed samples, the presence of *P*. *salmonis* was detected with Ct from 17 to 24 (Table 13).

**Table 13:** RT-PCR SRS results from necropsy of mortality of Atlantic salmon challenge with *P*. *salmonis* during the experiment.

Tank	Group	Fish (N°)	Ct	Prevalence SRS (%)
**2**	Challenge	1	20.73	100
**2**	Challenge	2	23.18	100
**3**	Challenge	1	17.44	100
**3**	Challenge	2	21.32	100
**5**	Positive control	1	24.06	100
**5**	Positive control	2	20.95	100
**6**	Positive control	1	17.27	100
**6**	Positive control	2	17.24	100

The treatment used here was successful in reducing mortality in the challenge group, when compared to the control group (without medication). Therefore, it can be suggested that oral administration of 20 mg/Kg bw of florfenicol in micro-rations for 15 days, increased the survival of the fish exposed to *Piscirickettsia salmonis* LF-89, displaying differences in the reduction of mortality and feed consumption compared to the group without medication.

All samples of kidney and liver, from tanks of challenge fish and positive control, were negative for *P*. *salmonis* (RT-PCR SRS) after treatment with florfenicol 20 mg/KG bw for 15 days (Table 14).

**Table 14:** RT-PCR SRS results from necropsy of euthanized fish (Atlantic salmon) after the experiment.

Tank	Sample ID	Matrix	SRS	Ct
**2**	1	Kidney	Negative	No Ct
	2	Liver	Negative	No Ct
**3**	1	Kidney	Negative	No Ct
	2	Liver	Negative	No Ct
**5**	1	Kidney	Negative	No Ct
	2	Liver	Negative	No Ct
**6**	1	Kidney	Negative	No Ct
	2	Liver	Negative	No Ct

## Discussion

It has been recently observed that the doses of florfenicol indicated in veterinary recommendations for the treatment of *P*. *salmonis* range from 20 to 40 mg/Kg l.w. [5], even though the Veterinary Medical Registry recommends a dose of 10 mg/kg l.w. for the different salmonid species. These doses have no scientific basis, considering that there is neither a clinical cut-off value nor an epidemiological cut-off point indicated by international organizations, such as EUCAST, VETCAST or CLSI, or national agencies responsible for monitoring bacterial resistance programs.

In order to assess whether an increase in florfenicol doses is associated with changes in phenotypic susceptibility, this study evaluated the MIC of 87 isolates of *P*. *salmonis* against florfenicol. The MIC ranges found were low, between 0.06 and 0.25 μg mL^-1^, which is consistent with those described by other researchers in the country [21, 49]. These results indicate that the susceptibility of strains of *P*. *salmonis* to florfenicol has remained steady over the years of the study, independent of geographical distribution or species of origin. This suggestion differs from other authors who have found variations in the patterns of *in vitro* antimicrobial sensitivity of *P*. *salmonis* isolates among different salmon species and geographical areas [50]. Moreover, other researchers in Chile found bimodal MIC distributions, overlapping wild-type and non-wild-type populations of *P*. *salmonis*, with fully susceptible and reduced susceptibility sub-populations [20, 33]. Our results suggest that only one population is present among these isolates, being fully susceptible to florfenicol.

By determining the MICs, we were also able to obtain the clinical pharmacodynamic value that we use in the PK/PD indices. The strains of *P*. *salmonis* showed a unimodal distribution and the CO_WT_ obtained was <0.25 μg mL^-1^ with a standard deviation of 0.47 log_2_ μg mL^-1^ and 0.358 log_2_ μg mL^-1^, independent of the statistical method used. The standard deviations are below the one described by the NRI of 1.2 log_2_ μg mL^-1^, showing that the CO_WT_ value obtained is accurate and valid. According to these results, all the strains analyzed were WT, indicating that they are susceptible to treatment with florfenicol [51].

On the other hand, considering that most susceptibility studies and CO_WT_ values are carried out in small bacterial populations, the PD value used in this research was two dilutions higher than the CO_WT_ obtained (1 μg/mL).

The PK/PD index used was AUC_0-24h_/MCI, considering that florfenicol belongs to the same family as thiamphenicol with concentration-independent activity and prolonged post-antibiotic effect [31]. This index has also been used in PK/PD studies of florfenicol carried out in other animal species against different pathogenic bacteria [34, 35].

In this scientific work, the PK/PD index (AUC_0-24h_ / MIC) was 141 ± 45, 166 ± 34 and 520 ± 50 for the doses of 10, 15 and 20 mg/Kg (Table 9), respectively. Since the values themselves do not indicate the probability of therapeutic success, the Monte Carlo simulation was needed to establish the dose that would allow a bacteriological cure and clinical response with a probability (PTA) > of 90%. The Monte Carlo simulation considers the variability of the PK and PD parameters, where a distribution of values is described and associated with a probability of inhibiting the microorganism (probability of therapeutic success) [30, 52].

According to the results of the Montecarlo simulation, a dose of 20 mg/Kg l.w. administered orally through feed has a 100% probability (PTA) of maximizing therapeutic success in infections caused by *P*. *salmonis* in salmonids. At this dose, plasma concentrations remained over the defined pharmacodynamic value (1 ±g mL) for 24 hours (AUC_0-24h_). Doses greater than those recommended as optimal could generate negative impact on the environment and promote bacterial resistance.

According to these results, we could also suggest that, at the indicated dose, the PK/PD cut-off point for florfenicol versus *P*. *salmonis* could be 2 ±g mL (PTA = 99%). Bacterial isolates that present MIC values higher than the described PK/PD cut-off point could be considered resistant from a clinical point of view.

During the challenge of Atlantic salmon (*Salmo* salar) with *P*. *salmonis*, behavior and cumulative mortality was in line with expectations (50–60%) and similar to previous calculations performed when determining LD_50_ (30-day evaluation). During evaluation of the main challenge, a clear difference was observed between the challenged group and the control group in terms of behavior, response to feeding, and mortality of individuals, (Figs 4 and 5). The feed consumption of the challenged fish remained constant during and after treatment. In terms of mortality, the treatment was successful in reducing mortality in the challenge group, when compared to the control group (without medication). Thus, it can be suggested that oral administration of 20 mg/Kg bw of florfenicol in micro-rations for 15 days, increased the survival of the fish exposed to *Piscirickettsia salmonis* LF-89, displaying differences in the reduction of mortality and feed consumption compared to the group without medication.

In conclusion, the results of the PK/PD studies and the Montecarlo simulation show that florfenicol at a dose of 20 mg/Kg bw has a high probability of therapeutic success. The *in vivo* studies show that florfenicol at the mencionated dose, administered for 15 consecutive days in micro-rations, is effective in reducing mortality and clinical signs from *Piscirickettsia salmonis* in Atlantic salmon (*Salmo salar*), and is the treatment of choice for this disease in farmed salmon.

Finally, good veterinary practice in the use of antimicrobials in food-producing animals, including farmed salmon, mandates selective use of antimicrobials in accordance with instructions for use of veterinary drugs registered by the regulatory authorities and indicates the need to respect mandatory withdrawal periods once treatment ends. The mandatory withdrawal period is a critical factor defined as the time during which a drug must not be administered prior to the slaughter of the animal for consumption [53]. The use of higher-than-optimal doses can lengthen these withdrawal periods, thus delaying salmon harvest.

## Supporting information

S1 Fig Integration of pharmacokinetic and pharmacodynamic variables (PK/PD) using the Montecarlo simulation. MIC = 1 μg/mL. a) Dose = 10 mg/Kg; b) Dose = 15 mg/Kg; b) Dose = 20 mg/Kg.
(PDF)

S1 Table Minimum Inhibitory Concentration values of 87 strains of *Piscirickettsia salmonis* versu*s* florfenicol. (PDF)

S2 Table Average plasma concentrations (μg/mL) by sampling time (hours) and dose: 10, 15 and 20 mg/Kg. (PDF)

S3 Table Pharmacokinetics parameters of florfenicol by the trial dose: 10, 15 and 20 mg/Kg. (PDF)

S4 Table Specific Feed Rate (%) by date of experiment and group. (PDF)

S5 Table Cumulative mortality (%) of challenge group by date of experiment. (PDF)

S6 Table Cumulative mortality (%) of control (+) group by date of experiment. (PDF)

S7 Table Cumulative mortality (%) of control (-) group by date of experiment. (PDF)
