# Supplementary material for: Optimization of florfenicol dose against Piscirickettsia salmonis in Salmo salar through PK/PD studies
Source: PLoS One. 2019 May 13;14(5):e0215174. doi: 10.1371/journal.pone.0215174 (PMC6513110; doi:10.1371/journal.pone.0215174)
Supplement: S1 Fig — MIC = 1 μg/mL. a) Dose = 10 mg/Kg; b) Dose = 15 mg/Kg; b) Dose = 20 mg/Kg. (PDF) [file pone.0215174.s001.pdf]

a)

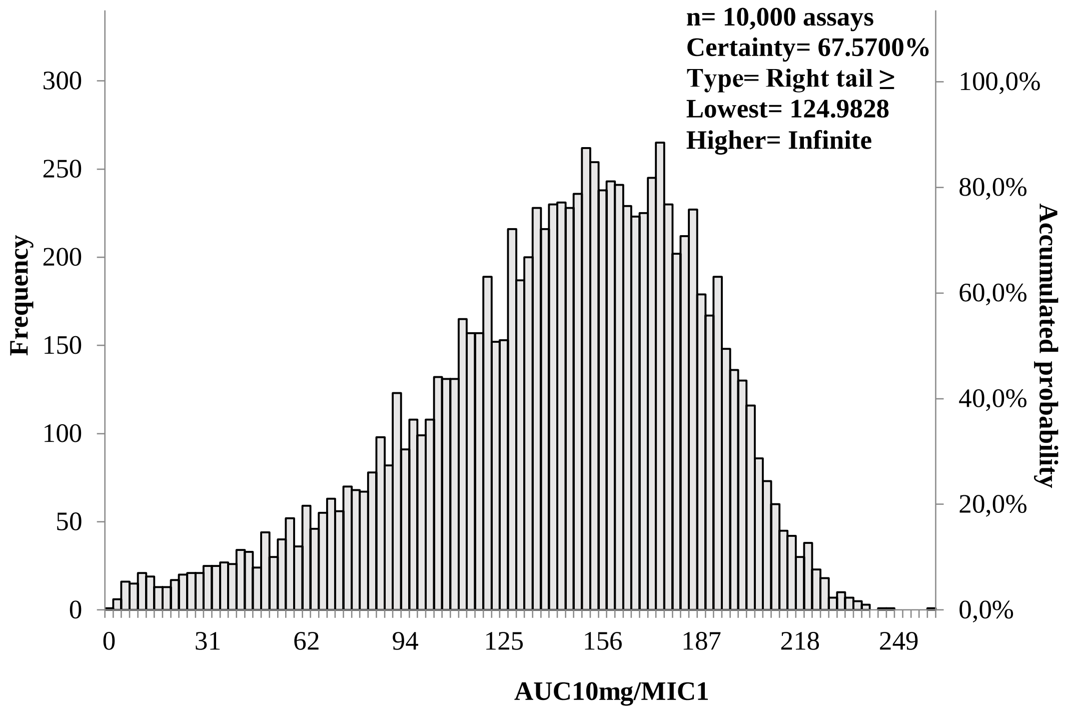
n= 10,000 assays
Certainty= 67.5700%
Type= Right tail ≥
Lowest= 124.9828
Higher= Infinite
Frequency
Accumulated probability
AUC10mg/MIC1
0
50
100
150
200
250
300
0,0%
20,0%
40,0%
60,0%
80,0%
100,0%
0
31
62
94
125
156
187
218
249

b)

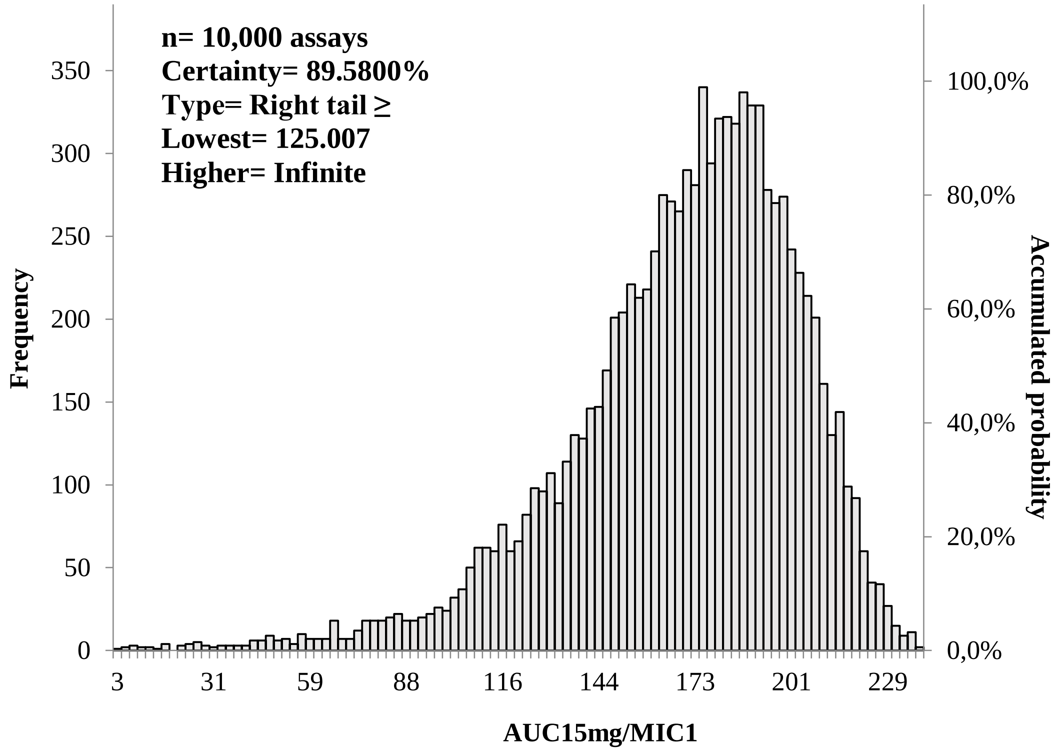
n= 10,000 assays
Certainty= 89.5800%
Type= Right tail ≥
Lowest= 125.007
Higher= Infinite
Frequency
Accumulated probability
AUC15mg/MIC1
0
50
100
150
200
250
300
350
0,0%
20,0%
40,0%
60,0%
80,0%
100,0%
3
31
59
88
116
144
173
201
229

c)

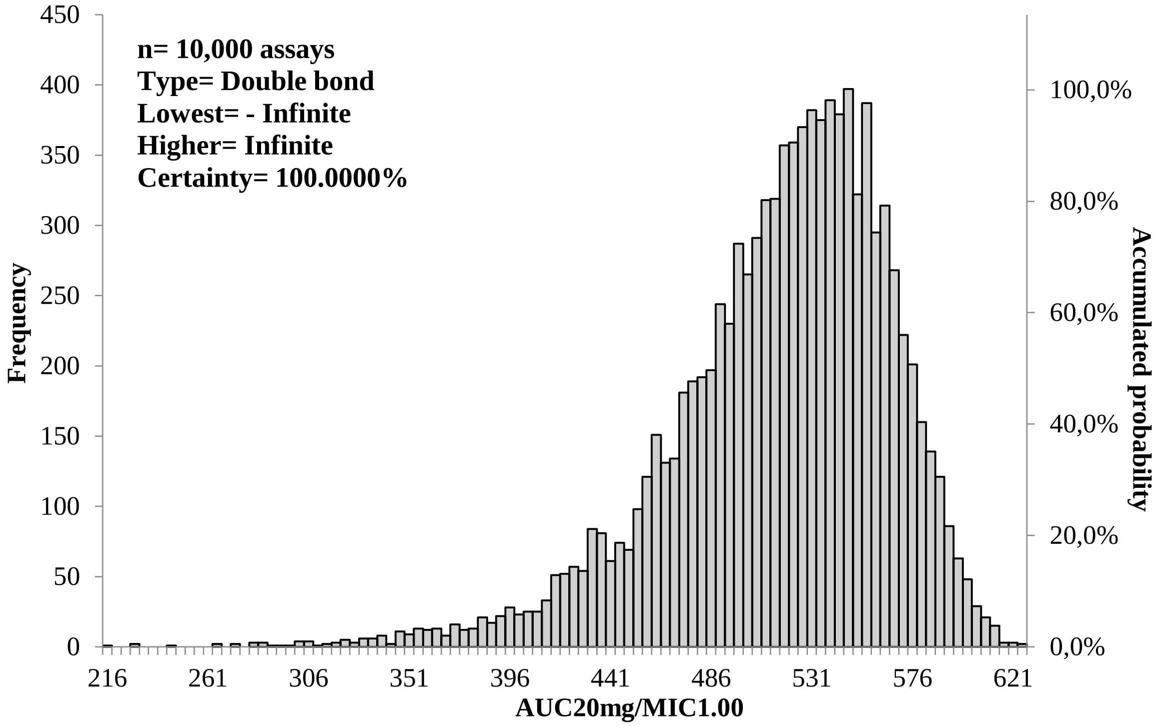

S1 Fig. Integration of pharmacokinetic and pharmacodynamic variables (PK/PD) using the Montecarlo simulation. MIC=1 µg/mL. a) Dose=10 mg/Kg; b) Dose=15 mg/Kg; b) Dose=20 mg/Kg.
