# Supplementary material for: Optimization of florfenicol dose against Piscirickettsia salmonis in Salmo salar through PK/PD studies
Source: PLoS One. 2019 May 13;14(5):e0215174. doi: 10.1371/journal.pone.0215174 (PMC6513110; doi:10.1371/journal.pone.0215174)
Supplement: S1 Table — (PDF) [file pone.0215174.s002.pdf]

S1 Table. Minimum Inhibitory Concentration values of 87 strains of *Piscirickettsia salmonis* versus florfenicol.

Sample ID	MIC
22	0.125
222	0.125
888	0.125
891	0.125
898	0.125
908	0.125
1224	0.125
1278	0.125
1279	0.25
1280	0.125
1281	0.125
1282	0.125
1287	0.06
1303	0.125
1315	0.125
1316	0.125
1317	0.125
1318	0.125
1319	0.125
1320	0.125
1321	0.125
1363	0.125
2192	0.125
2493	0.125
2494	0.125
2855	0.125
3045	0.125
5289	0.125
5449	0.125
5463	0.125
5549	0.125
6253	0.125
9386	0.125
9484	0.125

Sample ID	MIC
9734	0.125
10002	0.125
10197	0.125
10255-1	0.125
10255-2	0.125
10514	0.125
10534	0.125
10693	0.125
11152	0.125
11210	0.125
11216	0.125
11252	0.125
11259	0.125
11297	0.125
11330	0.125
11337	0.06
11531	0.125
11570	0.125
11608	0.125
11618	0.125
11742	0.125
11744	0.125
11755	0.125
11808	0.125
11942	0.125
11944	0.125
12093	0.125
12157	0.125
12181	0.06
12182	0.25
12282	0.06
12564	0.125
12565	0.125
12655	0.25

Sample ID	MIC
13287	0.125
14650	0.06
15677	0.125
17083	0.06
17139	0.125
17490	0.125
18059	0.125
18157	0.06
18380	0.125
18460	0.125
18446	0.125
18545	0.125
18627	0.125
18729	0.125
18756	0.25
18757	0.25
18874	0.25
18766	0.25
11337	0.06
11531	0.125
11570	0.125
11608	0.125
11618	0.125
11742	0.125
11744	0.125
11755	0.125
11808	0.125
11942	0.125
11944	0.125
12093	0.125
12157	0.125
12181	0.06

Sample ID	MIC
12564	0.125
12565	0.125
12655	0.25
13287	0.125
14650	0.06
15677	0.125
17083	0.06
17139	0.125
17490	0.125
18059	0.125
18157	0.06
18380	0.125
18460	0.125
18446	0.125
18545	0.125
18627	0.125
18729	0.125
18756	0.25
18757	0.25
18874	0.25
18766	0.25
11210	0.125
11216	0.125
11252	0.125
11259	0.125
11297	0.125
11330	0.125
12182	0.25
12282	0.06
