# Supplementary material for: Optimization of florfenicol dose against Piscirickettsia salmonis in Salmo salar through PK/PD studies
Source: PLoS One. 2019 May 13;14(5):e0215174. doi: 10.1371/journal.pone.0215174 (PMC6513110; doi:10.1371/journal.pone.0215174)
Supplement: S2 Table — (PDF) [file pone.0215174.s003.pdf]

S2 Table. Average plasma concentrations (µg/mL) by sampling time (hours) and dose: 10, 15 and 20 mg/Kg.

Dose (mg/Kg)	Fish	Sampling time (h)									
		T1 (0)	T2 (1)	T3 (3)	T4 (6)	T5 (8)	T6 (12)	T7 (16)	T8 (24)	T9 (36)	T10 (48)
10	1	0.00	0.00	0.68	3.36	8.74	0.70	0.09	0.60	0.31	0.02
	2	0.00	0.00	0.00	2.93	5.47	8.61	11.92	7.24	0.39	1.01
	3	0.00	0.07	0.00	4.73	5.39	0.18	7.98	9.16	0.97	0.87
	4	0.00	0.03	3.64	4.66	2.72	9.83	10.70	1.61	1.55	0.71
	5	0.00	0.00	2.10	2.80	1.40	15.5	2.1	1.5	1.6	0.8
	6	0.00	0.03	1.66	4.00	5.33	5.53	7.58	1.56	0.72	1.29
	7	0.00	0.00	1.20	2.50	6.60	12.5	7.5	2.0	1.6	0.6
	8	0.00	0.10	0.00	1.10	11.2	9.8	7.5	3.5	5.3	0.9
	9	0.00	0.20	2.00	0.90	5.3	10.7	7.0	3.2	0.4	0.7
	10	0.00	0.00	0.00	3.76	5.41	6.92	2.55	3.28	1.83	0.84
	11	0.00	0.10	3.37	0.55	1.91	9.83	7.49	4.15	1.72	0.78
	12	0.00	0.01	2.03	3.14	5.41	17.37	10.07	2.69	3.98	0.68
15	1	0.00	0.00	2.51	9.41	12.43	10.61	4.79	4.35	1.64	0.25
	2	0.00	0.20	5.39	12.40	11.76	9.57	6.44	3.74	1.64	0.60
	3	0.00	0.40	2.35	4.05	10.05	12.95	7.67	4.35	2.31	1.17
	4	0.00	1.35	2.38	8.04	13.11	10.28	4.48	2.63	2.46	0.15
	5	0.55	3.14	4.93	5.75	12.07	10.24	7.22	18.41	1.64	1.25
	6	0.00	0.00	3.69	5.90	8.14	14.66	6.28	1.96	2.52	0.07
	7	0.00	0.32	5.77	7.68	7.71	18.78	0.94	4.00	1.53	0.60
	8	0.00	0.00	3.01	3.92	11.94	2.50	2.89	7.01	0.77	0.39
	9	0.00	0.20	6.02	4.78	9.58	6.08	2.30	4.35	0.17	0.57
	10	0.00	0.00	5.95	5.53	7.78	10.26	6.41	1.97	3.31	0.60
	11	0.00	0.00	7.12	5.76	3.97	13.69	3.05	3.06	0.07	0.19
	12	0.00	1.25	6.70	6.66	12.11	3.72	2.98	3.35	1.64	1.54
20	1	0.00	0.00	0.30	8.61	8.23	26.18	20.94	19.80	11.62	0.00
	2	0.00	0.23	1.19	8.70	7.23	19.32	16.65	18.40	10.64	0.00
	3	0.00	0.00	1.99	4.52	6.99	18.31	21.56	17.49	11.29	0.00
	4	0.00	0.02	1.64	10.30	8.51	14.31	12.67	13.76	8.86	0.00
	5	0.00	0.00	0.76	8.04	7.91	20.25	17.12	17.98	9.83	0.01
	6	0.00	0.43	2.86	8.70	6.01	20.49	17.60	20.05	11.87	0.00
	7	0.00	0.48	4.17	10.50	6.99	16.23	18.70	17.94	10.64	0.00
	8	0.00	0.00	1.87	8.53	6.98	15.52	21.09	19.39	8.77	0.00
	9	0.00	0.00	0.53	8.70	6.99	20.49	17.13	16.71	11.29	0.00
	10	0.00	0.00	1.41	8.63	4.54	15.82	16.94	17.29	11.55	0.00
	11	0.00	0.00	1.66	10.48	6.35	18.38	15.27	16.63	10.64	0.00
	12	0.00	0.92	1.29	8.70	7.18	14.38	19.37	14.91	11.47	0.06
