# Supplementary material for: Optimization of florfenicol dose against Piscirickettsia salmonis in Salmo salar through PK/PD studies
Source: PLoS One. 2019 May 13;14(5):e0215174. doi: 10.1371/journal.pone.0215174 (PMC6513110; doi:10.1371/journal.pone.0215174)
Supplement: S3 Table — (PDF) [file pone.0215174.s004.pdf]

S3 Table. Pharmacokinetics parameters of florfenicol by the trial dose: 10, 15 and 20 mg/Kg.

**Dose (mg/Kg)**	**Fish**	**AUC**	**Cmax (µg/mL)**	**Tmax (h)**
**10**	1	51.00	8.73	8
	2	177.69	11.92	16
	3	100.72	9.16	24
	4	174.93	10.69	16
	5	127.02	15.51	12
	6	129.66	7.57	16
	7	170.63	12.52	12
	8	167.17	11.23	8
	9	146.57	10.67	12
	10	94.84	6.92	12
	11	133.29	9.83	12
	12	218.94	17.37	12
**15**	1	183.72	12.43	8
	2	201.37	11.76	8
	3	195.18	12.95	12
	4	178.27	13.10	8
	5	193.70	18.40	24
	6	194.01	14.66	12
	7	182.89	18.78	12
	8	102.14	11.93	8
	9	116.64	9.58	8
	10	169.46	10.25	12
	11	150.39	13.69	12
	12	125.26	12.11	8
**20**	1	621.44	26.17	12
	2	526.75	19.31	12
	3	536.74	21.56	16
	4	419.73	14.30	12
	5	531.17	20.24	12
	6	558.97	20.49	12
	7	528.33	18.70	16
	8	550.85	21.08	16
	9	515.36	20.48	12
	10	484.11	17.29	24
	11	491.37	18.37	24
	12	477.28	19.36	16
