# Supplementary material for: Optimization of florfenicol dose against Piscirickettsia salmonis in Salmo salar through PK/PD studies
Source: PLoS One. 2019 May 13;14(5):e0215174. doi: 10.1371/journal.pone.0215174 (PMC6513110; doi:10.1371/journal.pone.0215174)
Supplement: S4 Table — (PDF) [file pone.0215174.s005.pdf]

S4 Table. Specific Feed Rate (%) by date of experiment and group.

Date	SFR					
	**Days from challenge**	**Challenge group TK2**	**Challenge group TK3**	**Control (+) TK 5**	**Control (+) TK6**	**Control (-) TK 9**
**14-Aug**	0	0	0	0	0	0
**15-Aug**	1	0.73	0.61	0.67	0.60	0.77
**16-Aug**	2	0.90	0.84	0.86	0.70	0.92
**17-Aug**	3	0.84	0.97	0.77	0.91	0.96
**18-Aug**	4	1.09	0.99	1.23	1.19	1.22
**19-Aug**	5	1.16	1.08	1.27	1.19	1.23
**20-Aug**	6	1.19	1.06	0.97	1.15	1.21
**21-Aug**	7	1.22	1.10	1.06	1.32	1.11
**22-Aug**	8	1.22	111	1.23	1.40	1.02
**23-Aug**	9	1.22	1.17	1.18	1.36	1.12
**24-Aug**	10	1.08	1.09	1.08	1.26	1.46
**25-Aug**	11	1.29	1.26	1.47	1.21	1.48
**26-Aug**	12	1.18	1.08	1.34	1.21	1.46
**27-Aug**	13	1.15	1.03	1.27	1.19	1.45
**28-Aug**	14	1.28	1.20	0.60	1.23	1.12
**29-Aug**	15	0.99	1.03	0.38	0.79	1.35
**30-Aug**	16	1.21	1.18	0.86	0.78	1.44
**31-Aug**	17	1.22	1.13	0.92	0.89	1.45
**1-Sep**	18	1.29	1.28	1.29	0.73	1.42
**2-Sep**	19	1.27	1.21	1.04	0.94	1.42
**3-Sep**	20	1.17	1.15	1.13	1.21	1.50
**4-Sep**	21	1.37	1.33	1.18	1.09	1.34
**5-Sep**	22	1.39	1.36	1.13	1.09	1.24
**6-Sep**	23	1.39	1.37	1.25	1.14	1.50
**7-Sep**	24	1.42	1.45	1.33	1.19	1.50
**8-Sep**	25	1.45	1.49	1.29	1.19	1.50
**9-Sep**	26	1.43	1.40	1.26	1.11	1.43
**10-Sep**	27	1.44	1.40	1.16	0.99	1.41
**11-Sep**	28	1.43	1.46	1.34	1.17	1.50
**12-Sep**	29	1.44	1.46	1.34	1.25	1.46
**13-Sep**	30	1.46	1.45	1.29	1.12	1.50
**14-Sep**	31	1.44	1.43	1.39	0.98	1.35
**15-Sep**	32	1.48	1.47	1.42	1.02	1.50
**16-Sep**	33	1.46	1.46	1.40	0.86	1.38
**17-Sep**	34			1.31	0.82	1.45

**18-Sep**	35			1.47	1.47	1.50
**19-Sep**	36			1.38	1.38	1.50
**20-Sep**	37			1.15	1.33	1.50
**21-Sep**	38			1.10	1.29	1.47
**22-Sep**	39			1.20	1.42	1.50
**23-Sep**	40			1.50	1.50	1.44
**24-Sep**	41			1.42	1.39	1.41
**25-Sep**	42			1.40	1.46	1.45
**26-Sep**	43			1.42	1.44	1.50

Challenge group: Tanks 2 and 3; Control (+) (without medication): Tanks 5 and 6; Control (-) (without challenge): Tank 9. TK: Tank; SFR: Specific Feed Rate.
