# Supplementary material for: Optimization of florfenicol dose against Piscirickettsia salmonis in Salmo salar through PK/PD studies
Source: PLoS One. 2019 May 13;14(5):e0215174. doi: 10.1371/journal.pone.0215174 (PMC6513110; doi:10.1371/journal.pone.0215174)
Supplement: S5 Table — (PDF) [file pone.0215174.s006.pdf]

S5 Table. Cumulative mortality (%) of challenge group by date of experiment.

Date	Temperature (°C)	Day	Challenge group					
			Total (N°)	Mortality /day	Mortality (%)	Withdrawn /analysis	Accumulated Mort.	Mortality (%)
**14-Aug**	14.9	0	60	0.00	0.00	0.00	0.00	0.00
**15-Aug**	14.7	1	60	0.00	0.00	0.00	0.00	0.00
**16-Aug**	15.3	2	60	0.00	0.00	0.00	0.00	0.00
**17-Aug**	15.0	3	56	0.00	0.00	4.00	0.00	0.00
**18-Aug**	15.5	4	56	0.00	0.00	0.00	0.00	0.00
**19-Aug**	14.6	5	56	0.00	0.00	0.00	0.00	0.00
**20-Aug**	15.3	6	56	0.00	0.00	0.00	0.00	0.00
**21-Aug**	15.4	7	56	0.00	0.00	0.00	0.00	0.00
**22-Aug**	15.6	8	56	0.00	0.00	0.00	0.00	0.00
**23-Aug**	15.2	9	56	0.00	0.00	0.00	0.00	0.00
**24-Aug**	14.4	10	56	0.00	0.00	0.00	0.00	0.00
**25-Aug**	14.8	11	56	0.00	0.00	0.00	0.00	0.00
**26-Aug**	14.5	12	56	0.00	0.00	0.00	0.00	0.00
**27-Aug**	14.8	13	56	0.00	0.00	0.00	0.00	0.00
**28-Aug**	14.8	14	56	0.00	0.00	0.00	0.00	0.00
**29-Aug**	14.3	15	56	0.00	0.00	0.00	0.00	0.00
**30-Aug**	14.6	16	55	1.00	1.79	0.00	1.00	1.79
**31-Aug**	14.7	17	54	1.00	1.82	0.00	2.00	3.57
**1-Sep**	14.6	18	54	0.00	0.00	0.00	2.00	3.57
**2-Sep**	15.2	19	54	0.00	0.00	0.00	2.00	3.57
**3-Sep**	15.0	20	53	1.00	1.85	0.00	3.00	5.36
**4-Sep**	14.8	21	53	0.00	0.00	0.00	3.00	5.36
**5-Sep**	14.4	22	52	1.00	1.89	0.00	4.00	7.14
**6-Sep**	14.3	23	52	0.00	0.00	0.00	4.00	7.14
**7-Sep**	14.8	24	52	0.00	0.00	0.00	4.00	7.14
**8-Sep**	15.4	25	52	0.00	0.00	0.00	4.00	7.14
**9-Sep**	15.5	26	52	0.00	0.00	0.00	4.00	7.14
**10-Sep**	15.1	27	52	0.00	0.00	0.00	4.00	7.14
**11-Sep**	15.0	28	52	0.00	0.00	0.00	4.00	7.14
**12-Sep**	14.7	29	52	0.00	0.00	0.00	4.00	7.14
**13-Sep**	15.1	30	52	0.00	0.00	0.00	4.00	7.14
**14-Sep**	15.1	31	52	0.00	0.00	0.00	4.00	7.14
**15-Sep**	15.3	32	52	0.00	0.00	0.00	4.00	7.14
**16-Sep**	15.1	33	52	0.00	0.00	0.00	4.00	7.14
**17-Sep**	15.0	34						
**18-Sep**	15.0	35						
**19-Sep**	14.6	36						
**20-Sep**	14.8	37						
**21-Sep**	14.6	38						
**22-Sep**	14.7	39						
**23-Sep**	14.6	40						
**24-Sep**	14.8	41						
**25-Sep**	15.0	42						
**26-Sep**	14.8	43						

Mort: Mortality.
