# Supplementary material for: Optimization of florfenicol dose against Piscirickettsia salmonis in Salmo salar through PK/PD studies
Source: PLoS One. 2019 May 13;14(5):e0215174. doi: 10.1371/journal.pone.0215174 (PMC6513110; doi:10.1371/journal.pone.0215174)
Supplement: S6 Table — (PDF) [file pone.0215174.s007.pdf]

S6 Table. Cumulative mortality (%) of control (+) group by date of experiment.

Date	Temperature (°C)	Day	Control (+)					
			Total (N°)	Mortality /day	Mortality (%)	Withdrawn /analysis	Accumulated Mort.	Mortality (%)
14-Aug	14.9	0	60	0.00	0.00	0.00	0.00	0.00
15-Aug	14.7	1	60	0.00	0.00	0.00	0.00	0.00
16-Aug	15.3	2	60	0.00	0.00	0.00	0.00	0.00
17-Aug	15.0	3	60	0.00	0.00	0.00	0.00	0.00
18-Aug	15.5	4	60	0.00	0.00	0.00	0.00	0.00
19-Aug	14.6	5	60	0.00	0.00	0.00	0.00	0.00
20-Aug	15.3	6	58	0.00	0.00	2.00	0.00	0.00
21-Aug	15.4	7	58	0.00	0.00	0.00	0.00	0.00
22-Aug	15.6	8	58	0.00	0.00	0.00	0.00	0.00
23-Aug	15.2	9	58	0.00	0.00	0.00	0.00	0.00
24-Aug	14.4	10	58	0.00	0.00	0.00	0.00	0.00
25-Aug	14.8	11	58	0.00	0.00	0.00	0.00	0.00
26-Aug	14.5	12	58	0.00	0.00	0.00	0.00	0.00
27-Aug	14.8	13	57	1.00	1.72	0.00	1.00	1.72
28-Aug	14.8	14	53	4.00	7.02	0.00	5.00	8.62
29-Aug	14.3	15	52	1.00	1.89	0.00	6.00	10.34
30-Aug	14.6	16	48	4.00	7.69	0.00	10.00	17.24
31-Aug	14.7	17	44	4.00	8.33	0.00	14.00	24.14
1-Sep	14.6	18	38	6.00	13.64	0.00	20.00	34.48
2-Sep	15.2	19	37	1.00	2.63	0.00	21.00	36.21
3-Sep	15.0	20	35	2.00	5.41	0.00	23.00	39.66
4-Sep	14.8	21	32	3.00	8.57	0.00	26.00	44.83
5-Sep	14.4	22	30	2.00	6.25	0.00	28.00	48.28
6-Sep	14.3	23	29	1.00	3.33	0.00	29.00	50.00
7-Sep	14.8	24	29	0.00	0.00	0.00	29.00	50.00
8-Sep	15.4	25	29	0.00	0.00	0.00	29.00	50.00
9-Sep	15.5	26	25	4.00	13.79	0.00	33.00	56.90
10-Sep	15.1	27	25	0.00	0.00	0.00	33.00	56.90
11-Sep	15.0	28	25	0.00	0.00	0.00	33.00	56.90
12-Sep	14.7	29	23	2.00	8.00	0.00	35.00	60.34
13-Sep	15.1	30	23	0.00	0.00	0.00	35.00	60.34
14-Sep	15.1	31	23	0.00	0.00	0.00	35.00	60.34
15-Sep	15.3	32	22	1.00	4.35	0.00	36.00	62.07
16-Sep	15.1	33	22	0.00	0.00	0.00	36.00	62.07
17-Sep	15.0	34	22	0.00	0.00	0.00	36.00	62.07
18-Sep	15.0	35	22	0.00	0.00	0.00	36.00	62.07
19-Sep	14.6	36	21	1.00	4.55	0.00	37.00	63.79
20-Sep	14.8	37	21	0.00	0.00	0.00	37.00	63.79
21-Sep	14.6	38	21	0.00	0.00	0.00	37.00	63.79
22-Sep	14.7	39	21	0.00	0.00	0.00	37.00	63.79
23-Sep	14.6	40	21	0.00	0.00	0.00	37.00	63.79
24-Sep	14.8	41	21	0.00	0.00	0.00	37.00	63.79
25-Sep	15.0	42	21	0.00	0.00	0.00	37.00	63.79
26-Sep	14.8	43	21	0.00	0.00	0.00	37.00	63.79

Mort: Mortality.
