# Supplementary material for: Optimization of florfenicol dose against Piscirickettsia salmonis in Salmo salar through PK/PD studies
Source: PLoS One. 2019 May 13;14(5):e0215174. doi: 10.1371/journal.pone.0215174 (PMC6513110; doi:10.1371/journal.pone.0215174)
Supplement: S7 Table — (PDF) [file pone.0215174.s008.pdf]

S7 Table. Cumulative mortality (%) of control (-) group by date of experiment.

Date	Temperature (°C)	Day	Control (-)					
			Total (N°)	Mortality /day	Mortality (%)	Withdrawn /analysis	Accumulated Mort.	Mortality (%)
**14-Aug**	14.9	0	30	0.00	0.00	0.00	0.00	0.00
**15-Aug**	14.7	1	30	0.00	0.00	0.00	0.00	0.00
**16-Aug**	15.3	2	30	0.00	0.00	0.00	0.00	0.00
**17-Aug**	15.0	3	30	0.00	0.00	0.00	0.00	0.00
**18-Aug**	15.5	4	30	0.00	0.00	0.00	0.00	0.00
**19-Aug**	14.6	5	30	0.00	0.00	0.00	0.00	0.00
**20-Aug**	15.3	6	30	0.00	0.00	0.00	0.00	0.00
**21-Aug**	15.4	7	30	0.00	0.00	0.00	0.00	0.00
**22-Aug**	15.6	8	30	0.00	0.00	0.00	0.00	0.00
**23-Aug**	15.2	9	30	0.00	0.00	0.00	0.00	0.00
**24-Aug**	14.4	10	30	0.00	0.00	0.00	0.00	0.00
**25-Aug**	14.8	11	30	0.00	0.00	0.00	0.00	0.00
**26-Aug**	14.5	12	30	0.00	0.00	0.00	0.00	0.00
**27-Aug**	14.8	13	30	0.00	0.00	0.00	0.00	0.00
**28-Aug**	14.8	14	30	0.00	0.00	0.00	0.00	0.00
**29-Aug**	14.3	15	30	0.00	0.00	0.00	0.00	0.00
**30-Aug**	14.6	16	30	0.00	0.00	0.00	0.00	0.00
**31-Aug**	14.7	17	30	0.00	0.00	0.00	0.00	0.00
**1-Sep**	14.6	18	30	0.00	0.00	0.00	0.00	0.00
**2-Sep**	15.2	19	30	0.00	0.00	0.00	0.00	0.00
**3-Sep**	15.0	20	30	0.00	0.00	0.00	0.00	0.00
**4-Sep**	14.8	21	30	0.00	0.00	0.00	0.00	0.00
**5-Sep**	14.4	22	30	0.00	0.00	0.00	0.00	0.00
**6-Sep**	14.3	23	30	0.00	0.00	0.00	0.00	0.00
**7-Sep**	14.8	24	30	0.00	0.00	0.00	0.00	0.00
**8-Sep**	15.4	25	30	0.00	0.00	0.00	0.00	0.00
**9-Sep**	15.5	26	30	0.00	0.00	0.00	0.00	0.00
**10-Sep**	15.1	27	30	0.00	0.00	0.00	0.00	0.00
**11-Sep**	15.0	28	30	0.00	0.00	0.00	0.00	0.00
**12-Sep**	14.7	29	30	0.00	0.00	0.00	0.00	0.00
**13-Sep**	15.1	30	30	0.00	0.00	0.00	0.00	0.00
**14-Sep**	15.1	31	30	0.00	0.00	0.00	0.00	0.00
**15-Sep**	15.3	32	30	0.00	0.00	0.00	0.00	0.00
**16-Sep**	15.1	33	30	0.00	0.00	0.00	0.00	0.00
**17-Sep**	15.0	34	30	0.00	0.00	0.00	0.00	0.00
**18-Sep**	15.0	35	30	0.00	0.00	0.00	0.00	0.00
**19-Sep**	14.6	36	30	0.00	0.00	0.00	0.00	0.00
**20-Sep**	14.8	37	30	0.00	0.00	0.00	0.00	0.00
**21-Sep**	14.6	38	30	0.00	0.00	0.00	0.00	0.00
**22-Sep**	14.7	39	30	0.00	0.00	0.00	0.00	0.00
**23-Sep**	14.6	40	30	0.00	0.00	0.00	0.00	0.00
**24-Sep**	14.8	41	30	0.00	0.00	0.00	0.00	0.00
**25-Sep**	15.0	42	30	0.00	0.00	0.00	0.00	0.00
**26-Sep**	14.8	43	30	0.00	0.00	0.00	0.00	0.00

Mort: Mortality.
